# How Much Does HIV Positivity Affect the Presence of Oral HPV? A Molecular Epidemiology Survey

**DOI:** 10.3390/ijerph18178999

**Published:** 2021-08-26

**Authors:** Giuseppa Visalli, Angela Di Pietro, Monica Currò, Marianna Pruiti Ciarello, Flavia D’Andrea, Giuseppe Nunnari, Giovanni Francesco Pellicanò, Alessio Facciolà

**Affiliations:** 1 Department of Biomedical and Dental Sciences and MorphoFunctional Imaging, University of Messina, 98122 Messina, Italy; gvisalli@unime.it (G.V.); monica.curro@unime.it (M.C.); marianna.pruiti@gmail.com (M.P.C.); afacciola@unime.it (A.F.); 2 Department of Clinical and Experimental Medicine, Unit of Infectious Diseases, University of Messina, 98122 Messina, Italy; flavia.dandrea91@libero.it (F.D.); gnunnari@unime.it (G.N.); 3 Department of Human Pathology of the Adult and the Developmental Age “G. Barresi”, Unit of Infectious Diseases, University of Messina, 98122 Messina, Italy; giovanni.pellicano@unime.it

**Keywords:** HIV, sexual risk behaviors, oral HPV

## Abstract

HIV-positive people showed a high oral prevalence of HPV-DNA and have a greater incidence of head and neck carcinomas compared to general population. We performed a molecular survey evaluating the presence of HPV-DNA in saliva of HIV-positive and HIV-negative subjects in order to quantify the risk represented by HIV-positivity. The sample was made up by 102 subjects: 40 HIV-positive, 32 HIV-negative with sexual risk behaviors (SRB) and 30 HIV-negative without risk factors. DNA was extracted from cellular pellets and HPV detection and genotyping were performed by PCR assays. In the HIV-positive group (of which 58.3% declared SRB) 33.33% of the sample were HPV-positive (33.33% to high-risk genotypes, 25.0% to low-risk genotypes and 41.66% to other genotypes). In the HIV-negative SRB group, HPV-positive subjects were 37.04% (60.0% to high risk genotypes, 20.0% to low risk genotypes, and 20.0% to other genotypes). Finally, in the control group, the HPV-positive subjects were 7.14% (50% to high-risk genotypes and 50% to low-risk genotypes). In the HIV group, concerning the HPV positivity, there was no significant difference between subjects with and without SRBs. In summary, we found a high oral HPV-DNA detection in HIV+ group, showing a strong relationship between HIV and HPV.

## 1. Introduction

Human Papilloma Viruses (HPV) and Human Immunodeficiency Virus (HIV) are both sexually transmitted viruses causing chronic infections with a significant burden worldwide [1,2,3] and characterised by a high negative impact on public health and individual social life [4,5,6]. Particularly, HPV infection is considered the most common Sexually Transmitted Infections (STIs) both among men and women and it is a highly prevalent STDs in people living with HIV (PLHIV) [7,8]. There are more than 100 different HPV-genotypes, grouped according their capacity to cause benign or malignant lesions in “low-risk HPV” (Lr-HPV), including types 6, 11, 42, 43 and 44, and “high-risk HPV” (Hr-HPV), including the genotypes 16, 18, 31, 33, 35, 39, 45, 51, 52, 56, 58, 59, 66 and 68 [9,10]. These viruses are characterized by a wide body distribution but they have been detected especially in cervical, ano-genital and oral lesions [11]. In general, HPVs are associated with more than 90% of anal and cervical cancers, about 70% of oropharyngeal, vaginal and vulvar cancers, more than 60% of penile cancers, and more than 10% of oral cavity cancers [12]. Based on GLOBOCAN data, about 2,2 million cancer cases (13% of global cancer incidence) are attributable to infections and HPV contributes for 690,000 new cases worldwide, including also ano-genital cancers [13,14,15,16,17,18,19,20].

Of all the head and neck cancers, Oral Squamous Cell Carcinoma (OSCC) is the most common oral cancer accounting for the 90% of all oral malignancies; this cancer represents approximately 5% and 2% of all malignancies in men and women respectively [21]. At least 90% of HPV-positive OSCCs are associated with HPV16 [22,23,24] and it has been showed that oral sex plays an important role in their acquisition [25,26]. The prevalence of the infection is eightfold higher among sexually experienced individuals and increases significantly with number of sexual partners [27]. Moreover, lifetime number of oral sexual partners is considered to be the behavioral factor most strongly associated with oropharyngeal cancer [28,29]. However, the risk of infection by sexual contact is multifactorial and it is influenced by both the number of sexual partners during a lifetime and the type of sexual contacts. Indeed, a recent study investigated a novel behavioural picture of HPV-related oropharyngeal cancer (sexual debut behaviors, exposure intensity, and relationship dynamics) and suggested timing and intensity of oral sex as novel independent risk factors [30]. Men who have sex with men (MSM) are at elevated risk of HPV acquisition and subsequent development of HPV cancers caused of their high-risk sexual behaviors [31]. In fact, MSM and women who have sex with women have a high prevalence of oral HPV infection compared to the whole of population [32,33].

The introduction of the highly active antiretroviral therapy (HAART) in the treatment of the HIV infection has significantly modified the natural history of this disease increasing the survival and reducing the mortality for AIDS-related diseases. However, the increased survival has enhanced, in these patients, the morbidity and mortality from chronic disorders and malignancies [34,35,36,37,38,39].

To date, there are around 38 million of HIV-positive adults and children, of which 26.0 million had access to HAART [40]. PLHIV are at increased risk of cancer compared with general population [41]. Indeed, nowadays, cancers have become the leading cause of mortality in HIV-positive people [42], not only for AIDS-defining malignancies (ADMs), but also for some non-AIDS-defining malignancies (NADMs) [43]. Among non-AIDS cancers, HPV-associated malignancies received great attention because of their multi-site distribution (especially head and neck and ano-genital tracts) and availability of preventive measures such as vaccination and screening strategies.

The oropharyngeal compartment is central to the persistence of HPV, and the virus is commonly detected in the oral mucosa of HIV-positive patients compared to HIV-negative ones. It has been established that HIV-positive people have a 3-fold higher standardized incidence of head and neck carcinomas compared to general population [44] and that, in these subjects, about the half of the OSCCs are HPV-related [45]. Moreover, HIV-positive people resulted 2.1 more likely to harbor HPV in the oral cavity compared to HIV-negative subjects [46]. Epidemiological evaluations carried out on HIV-infected people showed an overall oral prevalence of HPV-DNA ranging between 20% and 45%, with the type HPV16 found in a percentage between 12 and 26% [47,48]. In a study conducted on 90 MSM, the prevalence of HPV types covered by the nonavalent vaccine was 77.8% in HIV-positive subjects and the HPV16 and HPV58 were the mostly detected serotypes [49]. In a recent meta-analysis conducted on MSM, the prevalence of any HPV types on the oral cavity was 17.1% in HIV-negative and 28.9% in HIV-positive MSM [50]. In HIV-infected people, there are several factors favoring an oral HPV infection among which the same HIV infection, a severe immunodepression and a high number of sexual partners are the most important [50,51].

The improvement of the knowledge about the prevalence of HPV types covered by the newest nonavalent vaccine-in at risk categories of subjects would provide some relevant information about the number of people that hypothetically could be protected with the implementation of HPV vaccination. To this aim, we conducted a molecular epidemiological survey evaluating the presence of HPV-DNA in saliva samples of HIV-positive and HIV-negative subjects and therefore quantifying the risk represented by the HIV-positivity condition.

## 2. Materials and Methods

### 2.1. Patients

The study enrolled 102 subjects divided in three groups, 40 HIV-positive subjects (HIV+ group) with and without SRB, 32 HIV-negative subjects with sexual risk behaviors (SRB group) and 30 HIV-negative subjects without risk factors (control group). Specifically, we considered SRB a sexual promiscuity (>1 sexual partner), changing sexual partner frequently and the occurrence of unprotected sexual intercourse. The HIV+ group was made up by patients followed by the Infectious Diseases Operative Unit of the Messina University Hospital “G. Martino”. All the subjects were under HAART treatment and all have CD4+ count >200/mm^3^ (mean 640.89 ± 268.80). Moreover, concerning the HIV viral load, while 68.57% were Target Not Detectable (TND), 31.43% had a circulating viral load of which the mean value was 1577 ± 1441 copies/mL. The SRB group included subjects who spontaneously came to the HIV laboratory of the same University Hospital to undergo the HIV screening test after SRBs. The control group was selected among healthcare workers undergoing the routine health surveillance, after ascertaining the conditions necessary for enrollment in the aforementioned group. Specifically, these conditions were the HIV-negativity, the absence of SRBs (a single partner for at least three years) and of previous HPV diagnosis. After having received their written informed consent, an anonymous questionnaire was administered in order to collect information about socio-demographical data (age, gender, education level) and sexual habits.

### 2.2. Samples Collection

For the collection of saliva samples, we asked to participants to discarded the first spit to eliminate food debris and unwanted substances possibly contaminating the sample. Then, we asked participants to briefly refrain from swallowing (for 30 s) and spit much saliva was in the mouth with a single spit into a pre-labelled sterile container to collect about 2 mL of saliva. The samples were immediately refrigerated (+4 °C) to minimize degradation until further processing.

### 2.3. DNA Extraction

Oral samples were centrifuged at 3000 g for 10 min at 4 °C, the supernatant was removed and the pellet was resuspended in 10 mL of sterile saline solution; the method was repeated and the obtained pellets were stored at −80 °C until DNA purification. DNA was extracted from cellular pellets using Puregene DNA purification system (Qiagen, Milan, Italy) according to manufacturer’s instructions. DNA concentration and quality were estimated by spectrophotometer measurements of absorbance at 260 and 280 nm and electrophoresis (BioPhotometer plus, Eppendorf, Hamburg, Germany).

### 2.4. Detection of HPV-DNA

HPV detection in saliva DNA samples were, first to all, carried out by a PCR assay using the consensus primers MY09/MY11, which amplify a fragment of 450 bp within the L1 gene region of the viral genome. Subsequently, HPV-positive samples were amplified with a set of seven different-HPV-type specific primers. The sequences of primers used and the PCR profile were those of our previous study [9]. Each PCR run was monitored to contamination and overall end point sensitivity including a negative (sterile waters) and a positive control (DNA sample of an HPV type 16 carrier). In parallel, to verify DNA integrity, each sample was amplified for β-globin (268 bp). Amplification was performed in a Hybaid PCR sprint thermocycler and the PCR products were analyzed by 2% agarose gel electrophoresis, stained with ethidium bromide and visualized with ultraviolet transilluminator.

### 2.5. Statistical Analyses

Statistical analyses were performed using Prism 4.0 software (GraphPad, San Diego, CA, USA). The association between HPV oral infection and socio-clinical variables was assessed using chi-square tests, evaluating the Odds Ratio (OR) and the 95% Confidence Interval (CI). Correlations were determined using the Spearman′s rank correlation test. A multivariate regression analyses was performed using a priori model to assess the role of the socio-demographic, behavioral and clinical variables in the HIV+ group. Significance was assessed at the *p* < 0.05 level.

## 3. Results

Not all the saliva specimens of the subjects enrolled in our study presented a sufficient concentration of DNA. Therefore, the search for the HPV was made only in 90.2% of the initial sample. In particular, the specimens were collected from 36 HIV-positive subjects (HIV+ group), aged between 22 and 59 years old (39.95 ± 10.86), of which 58.33% with SRBs, 28 HIV negative subjects with SRB (SRB group), aged between 19 and 51 years old (28.17 ± 8.33), and 28 healthy subjects without SRBs (control group), aged between 25 and 54 years old (34.33 ± 10.29). The considered demographic and behavioral variables are shown in Table 1.

The concentration of DNA isolated from all the collected saliva samples was between 100 and 300 ng/μL. Absorbance measurements and A260/A280 ratio analysis confirmed the purity of the DNA isolated, which averaged between 1.7 and 2.0.

**Figure 1:**
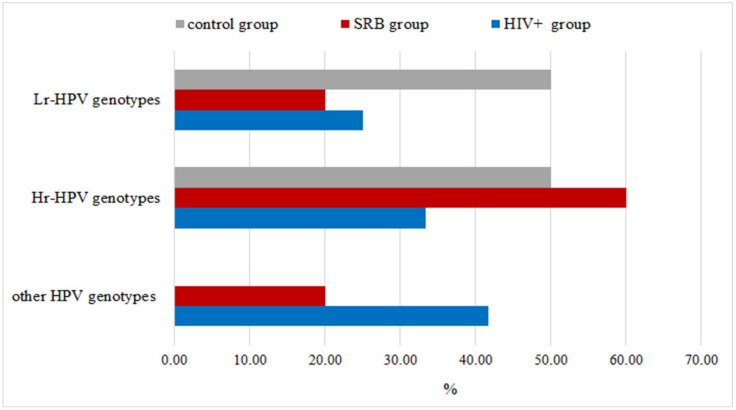
Percentage of oral HPV-positivity in the three considered groups.

As shown in Figure 1, in HIV+ group 12 samples (33.33%) were HPV-positive, of which 6 detected in HIV+ subjects with SRB and 6 in HIV+ subjects without SRB. Among the positive samples, 4 (33.33%) were positive to investigated Hr-HPV genotypes, 3 (25.0%) to Lr-HPV genotypes and 5 (41.66%) to other genotypes. In the SRB group, HPV-positive subjects were 10 (35.71%), of which 6 (60.0%) were positive to investigated Hr-HPV-genotypes, 2 (20.0%) to Lr-HPV genotypes, and 2 (20.0%) to other genotypes. Finally, in the control group, the HPV-positive subjects were 2 (7.14%), of which 1 was positive to Hr-HPV genotypes and 1 to Lr-HPV genotypes.

**Figure 2:**
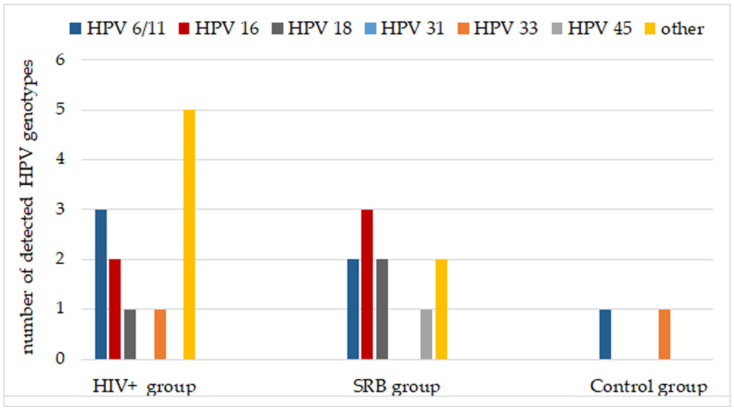
Genotype characterization of the HPV-positive samples.

The Figure 2 shows the genotype characterization of HPV-positive in the three examined groups. HPV 6/11 was the most frequent type (29.17%) followed by the HPV16 (20.83%), HPV18 (12.50%), HPV33 (8.33%) and then by HPV45 (4.17%). The percentage of positivity to other HPV genotypes was 29.17%.

**Figure 3:**
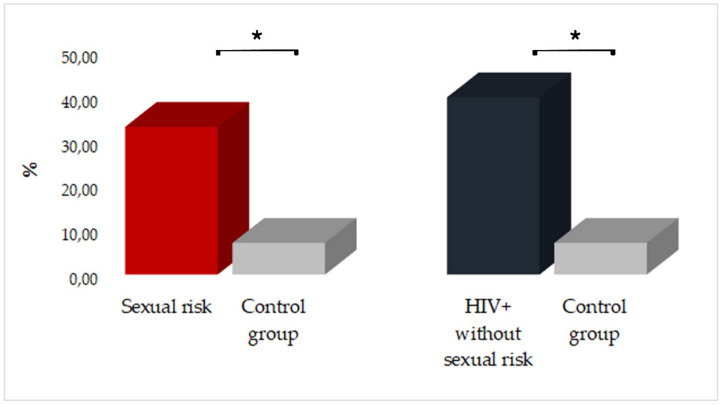
Assessment of SRBs (SRB group and HIV+ subjects with SRBs) and HIV-positivity as factors promoting HPV-infection (* = *p* < 0.05).

To investigate in the HPV+ subjects a potential risk factor promoting the presence of the virus in the saliva, we correlated these data with the collected information. Specifically, there was no associations between the HPV positivity and the socio-demographic characteristics while SRB and HIV-positivity were significantly associated to the presence of HPV-DNA in saliva. In particular, as shown in Figure 3, analyzing the data of HPV-DNA in saliva, we revealed that the variable “sexual risk” present in SRB group and in HIV+ subjects with SRB was strongly related to the oral HPV infection (*p* = 0.011; OR = 6.500; 95% CI: 1.368–30.8903). Similarly, comparing the control group respect HIV+ without SRB, we found that also HIV positivity was a condition promoting oral HPV infection (*p* = 0.014; OR = 8.667; 95% CI: 1.474–50.940).

In the HIV+ group, the multivariate analyses showed that, among the considered variables, only the HIV viral load was significantly related to the presence of oral HPV (*p* = 0.029) while no effects were shown by the SRB and socio-demographic variables (Table 2). The presence of circulating HIV as a promoting factor for oral HPV presence was confirmed by the OR obtained comparing HIV subjects with not detected viral load and HIV subjects with not suppressed viral load (*p* = 0.0152; OR = 8.75095% CI: 1.711–44.740).

## 4. Discussion

HPV infection is associated with several risk factors [52,53,54,55], among which HIV infection and other STIs. Specifically, the burden of HPV infection and cervical cancer is three times higher in HIV-positive women [56] compared to negative ones [57] as well as the presence of Hr-HPV serotypes (48.4% vs.17.3%) [58].

Furthermore, the incidence of HPV-associated head and neck cancer is increased in HIV-infected people, who are about six times more at risk to develop this kind of cancers than HIV-uninfected individuals [50]. At the same time, the incidence of HPV-associated anal and cervical cancer is 80 and 22× higher, respectively, in HIV-infected individuals than in HIV-uninfected ones [59]. Several studies show that HIV infection is associated with higher LSIL (low-grade squamous intraepithelial lesion) incidence compared to HSIL (high-grade squamous intraepithelial lesion) and higher prevalence of Lr-HPV types (3.6 to 5.6 times in HIV-seropositive women compared to HIV seronegative) [60,61,62].

Evidences show that especially the first step of infection characterized by the entry of the virus thanks to a disruption of the epithelium and its penetration into the basal epithelial layer, could be affected by direct and indirect interactions between HIV and HPV within the epithelial cells. This suggests that immunosuppression induced by HIV may play a greater role in the earlier phases of HPV natural history, i.e., acquisition, persistence, and progression to low-grade lesions, while it seems to less affect the later stages of carcinogenicity [63,64,65,66]. However, immunocompromise is not the only possible mechanism to explain the higher incidence of HPV in HIV-positive subjects.

Our study considered different groups of subjects and aimed to highlight the relative influence of the studied variables (HIV positivity and SRB). While the well-known role played by SRB in the acquisition of oral HPV has been confirmed in this study, our results emphasize the role of the HIV infection in HPV oral prevalence independently by the presence of SRB. Indeed, the results, showing the absence of significant differences between the HIV+ and SRB groups, seems to indicate an equal weight between SRB and HIV-positive status. Moreover, if we consider only the HIV+ group and discriminate it on the basis of the variable SRB, there is no difference in the prevalence of HPV. This result, along with the positive correlation between HPV positivity and HIV viral load, allow us to hypothesize various possible scenarios. SRB, as is well-known [67], certainly represents a factor favoring the HPV infection and our results confirm this association. However, the condition of HIV positivity, even in the absence of SRB, represents a variable favoring oral HPV infection [50].

As we have already said, HPV acquisition is increased in HIV-positive immunosuppressed, but, alternatively, the high HPV detection rates could be due to an increased HPV replication and/or persistence rather than an increased HPV acquisition and this increased persistence could explain the increased prevalence of oral warts in HAART treated HIV+ subjects [68]. Therefore, in HIV infected people, pharmacological treatment of the infection, rather than immunosuppression, potentially plays a role in HPV infections [68].

In our study, considering that all the investigated HIV-positive patients had a CD4+ number >200/mm^3^, we cannot attribute to the immunecompromise the explanation for a higher incidence of HPV infection, but rather we can hypothesize that other HIV-dependent mechanisms, such as a high viral load on the one hand and antiretroviral therapy on the other, are involved.

The higher prevalence of HPV infection that we found in HIV+ subjects with detectable viral load underlines a direct role of HIV, as confirmed by several studies [59,69,70,71,72,73,74,75] showing that the viral proteins gp120 and tat and the HIV-associated activation of inflammatory processes in the mucosal epithelium, can lead to the disruption of epithelial junctions, which may promote the penetration and/or dissemination of other viruses, including herpesviruses (HSV) and HPV.

HIV-associated disruption of mucosal epithelium may occur from initial phases of infection and it is present during systemic HIV/AIDS disease. Particularly, in subjects with high viral load, the infiltration of HIV-infected CD4+ lymphocytes, dendritic cells (DCs) and macrophages into the oral, intestinal and genital mucosa determines the release of not only mature HIV virions but also of the viral proteins gp120 and tat, which interact with epithelial cells through HIV coreceptors and integrins. Specifically, the prolonged interaction of tat protein with oral epithelial cells for 3 to 5 days leads to activation of ERK1/2 MAPK and substantial disruption of ZO-1, occludin and claudin-1 [59]. Cytological analysis of oral and anal epithelia from HIV-infected subjects showed that approximately 60% of tissues have disrupted epithelial junctions with the loss of the epithelial barrier integrity [59].

The disruption of oral and anal epithelium in HIV-infected individuals mediated by the HIV proteins tat and gp120 represents a mechanism for determining HPV penetration into basal and parabasal layers of epithelial cells. This issue may contribute to the higher prevalence of anogenital and oral HPV infection, and ultimately, increased risk of HPV-associated neoplasia in these individuals. HIV-associated disruption of oral and genital epithelia facilitates penetration by HPV.

The effect of HAART on HPV-related disease is still unclear because an equal number of studies agree and not agree with an association between HAART and HPV pathogenesis. For example, one study found significantly lower incidence of HPV infection and cervical lesions in women who were virologically suppressed by HAART compared to women who were not on HAART or not virologically suppressed [76] (Minkoff). However, other studies show that prolonged use of HAART adversely affects mucosal turnover, with acquisition and establishment of oral disease [44,77,78]. These inconsistent results may be due to changes in HAART regimens and use over time. A longer duration on HAART is also associated with reduced HSIL incidence [79,80], which may indicate immune system restoration [81].

In contrast to other HIV-associated cancers, since the introduction of HAART, the incidence of HPV-associated malignancies has increased [82]. Several studies have shown that HAART had little effect in reducing the increased risk of anal HPV infection in both men and women [83,84]. Other researches have reported a positive effect on the clearance of cervical and anal HPV infection in HIV-infected women and men respectively since the starting of HAART but overall, the prevalence of both cervical and anal HPV infection remains high among HIV-infected individuals in the HAART era [85].

The hypothesis that the prolonged use of HAART adversely affects the turnover rate of the mucosa has been advanced, and this could favour acquisition and establishment of oral disease [44,77,78]. Indeed, HAART usage is associated with development of adverse oral complications, among which oral ulcerations, epithelial hyperplasia and xerostomia [77,78], damaging the epithelium and potentially exposing the underlying tissues to infections due to multiple microorganisms, including HPV [44,77,78]. One study suggested that HAART treatment could damage the barrier function of the oral epithelium, increasing invasiveness, and thus malignancy of the HPV-infection [67]. In support of these observations, it has been demonstrated that HIV infected patients with an undetectable HIV load had a six-fold risk of HPV oral lesions [86].

## 5. Conclusions

In summary, in this study, we found a high oral HPV-DNA detection in HIV+ group showing a strong relationship between these sexually transmitted viruses: HIV and HPV. The burden of disease associated with HPV infection, particularly from Hr-HPV types, was considerable both in HIV-negative individuals with SRBs, and in HIV-positive individuals with and without SRBs. Therefore, we believe it is fundamental to act through informative health education campaigns by implementing HPV vaccinations on all general population, as recommended by the Italian Vaccine National Plan. Moreover, it is important to highlight that most the infection detected in this study would have been avoided if the subjects were vaccinated, given that the new nonavalent vaccine includes these strains. Furthermore, the association of these sexually transmitted infections underlines the critical need of a specific prevention strategy in the HIV-positive population and people with SRB. Enhanced HPV vaccine-based prevention and education programs need to be implemented in this high-risk community, and as clinical trials of vaccine efficacy in preventing HPV-associated malignancies in HIV/AIDS patients are not yet available. Equally fundamental are screening programs aimed at identifying oral, cervical and anal precancerous and cancerous lesions, regardless of the status of HAART treatment, CD4 + T cell count and viral load.

## 6. Limitation of the Study

The study presents some limits. The control group was made up by 100% heterosexual. It is possible that there is a difference in engagement in oral sex in groups with different sexual habits which may partly attribute to differences in HPV infection. Moreover, we did not have information about some risk factors of oral HPV infection, including smoking, lifetime number of sexual partners, and the number of oral sex partners. Finally, the lack of difference in the prevalence of HPV in the HIV+ subjects with and without SRB could be affected by the small sample size.

## Figures and Tables

**Table 1:** Socio-demographic characteristics and sexual habits of the tested subjects.

	HIV+ Group		SRB Group	Control Group
	Without Sexual Risk	With Sexual Risk		
N° SAMPLES	15	28	21	28
GENDER				
Men	78.57%	85.71%	66.67%	65.32%
Women	21.43%	14.29%	33.33%	34.68%
MEAN AGE (DS)	41.36 (11.73)	38.25 (10.18)	28.17 (8.33)	34.33 (10.29)
min–max	24–59	22–59	19–51	25–54
NATIONALITY				
Italian	71.43%	95.24%	95.83%	96.43%
Foreigners	28.57%	4.76%	4.17%	3.57%
EDUCATIONAL LEVEL				
Elementary school	7.14%	0.00%	0.00%	0.00%
Middle school	28.57%	23.81%	4.17%	0.00%
High school	50.00%	42.86%	62.50%	21.43%
University	14.29%	33.33%	33.34%	78.57%
SEXUAL HABITS				
Heterosexual	64.29%	33.33%	54.17%	100%
Homosexual	21.43%	57.14%	33.33%	
Bisexual	14.28%	9.52%	12.50%	

**Table 2:** Multivariate regression analyses of the studied variables and the oral HPV presence in the HIV+ group (R2 = 0.286).

	β Value	Standard Error of β Value	*p* Level
Gender	0.101	0.182	0.584
Age	−0.349	0.189	0.078
Nationality	−0.282	0.170	0.111
Education level	−0.042	0.163	0.797
Sexual orientation	−0.053	0.204	0.798
SRB	−0.132	0.174	0.454
CD4+ count	0.203	0.170	0.245
HIV viral load	0.470	0.202	0.029

## Data Availability

Not applicable.
